# Association Between Clinical Frailty and Antibiotic Resistance Among Older Patients with Fever in the Emergency Department

**DOI:** 10.3390/antibiotics15030296

**Published:** 2026-03-14

**Authors:** Ji Yeon Lim, Dong Hoon Lee, Ho Sub Chung, Yunhyung Choi, Yoon Hee Choi, Keon Kim, Ki-Hun Hong, Sung Jin Bae

**Affiliations:** 1 Department of Emergency Medicine, Ewha Womans University Seoul Hospital, College of Medicine, Ewha Womans University, 260 Gonghang-daero, Gangseo-gu, Seoul 07804, Republic of Korea; jylim0923@ewha.ac.kr (J.Y.L.); mikky5163@ewha.ac.kr (K.K.); 2 Department of Emergency Medicine, Chung-Ang University Gwangmyeong Hospital, College of Medicine, Chung-Ang University, 110 Deokan-ro, Gwangmyeong-si 14353, Republic of Korea; emdhlee@cau.ac.kr (D.H.L.); hoshap@cauhs.or.kr (H.S.C.); yunhyung0710@cauhs.or.kr (Y.C.); hongk007@cauhs.or.kr (K.-H.H.); 3 Department of Emergency Medicine, Ewha Womans University Mokdong Hospital, College of Medicine, Ewha Womans University, 1071 Anyangcheon-ro, Yangcheon-gu, Seoul 07985, Republic of Korea; unii@ewha.ac.kr

**Keywords:** frailty, drug resistance, bacterial, aged, fever, emergency department

## Abstract

**Background**: Frailty may predispose older adults to antibiotic-resistant infections; however, evidence in emergency department (ED) patients with fever remains limited. **Methods**: We conducted a retrospective multicenter cohort study of 544 ED patients aged ≥65 years with fever (tympanic temperature ≥ 37.5 °C) between August and October 2023. The cohort included 234 men and 310 women. Frailty was assessed using the Clinical Frailty Scale (CFS) and categorized as robust (CFS 1–3), pre-frail (CFS 4–5), or frail (CFS 6–9). ED-initiated microbiological cultures were obtained in 329/544 (60.5%) patients. The primary outcome was the detection of antibiotic-resistant isolates among culture-tested patients. **Results**: Among culture-tested patients (*n* = 329), antibiotic-resistant isolates were detected in 65/329 (19.8%), with a graded increase across frailty strata: robust 13/121 (10.7%), pre-frail 16/88 (18.2%), and frail 36/120 (30.0%). In multivariable logistic regression restricted to culture-tested patients, frailty was independently associated with resistant infection (adjusted OR 2.84, 95% CI 1.15–7.04, *p* = 0.024). Frail patients also experienced greater therapeutic complexity, including higher rates of antibiotic regimen modification (68.1% vs. 54.5%) and longer antibiotic duration (median, 11 vs. 8 days), as well as worse clinical outcomes, including higher ICU admission (37.7% vs. 17.8%) and in-hospital mortality (7.2% vs. 1.8%) compared with robust patients. **Conclusions**: Frailty is independently associated with antibiotic-resistant infections in older ED patients with fever. Integrating frailty assessment into ED protocols can enhance risk stratification, inform empirical antibiotic selection, and antimicrobial stewardship strategies.

## 1. Introduction

Infectious diseases are a leading cause of morbidity and mortality among older adults presenting to emergency departments (EDs), accounting for approximately 15% of all ED visits in this population [1,2]. Managing these infections is particularly challenging because clinical presentations in older patients are often atypical and obscured by multiple comorbidities and polypharmacy. Moreover, age-related physiological changes significantly alter the pharmacokinetics and pharmacodynamics of antimicrobial agents, further complicating treatment decisions [3,4]. The rising prevalence of antibiotic-resistant pathogens further complicates this problem, as inappropriate empirical antibiotic therapy is associated with increased mortality, prolonged hospital stays, and higher healthcare costs [5,6]. Because older ED patients often present with overlapping clinical and healthcare exposure risk factors, additional clinically actionable markers are needed to better stratify the risk of resistant infection at the point of care.

Frailty, a state of increased vulnerability to adverse outcomes resulting from age-related decline in physiological reserves across multiple organ systems, has emerged as an important predictor of poor outcomes in older adults [7,8]. Emerging evidence suggests that frailty predisposes individuals to more severe infections through mechanisms involving immunosenescence, inflammaging (chronic low-grade inflammation in aging), and gut microbiome dysbiosis [9,10,11]. Among these mechanisms, inflammaging—characterized by elevated circulating pro-inflammatory cytokines such as IL-6, TNF-α, and C-reactive protein—creates a chronic inflammatory milieu that may impair immune surveillance [12,13]. Furthermore, frail older adults often exhibit gut microbiome dysbiosis, characterized by reduced microbial diversity and shifts in community composition [14,15,16]. This dysbiotic state, particularly during physiological stress or antibiotic exposure, may weaken colonization resistance and facilitate colonization by antibiotic-resistant pathogens [17,18,19]. However, it remains unclear whether frailty independently predicts antibiotic-resistant bacterial infections and influences antibiotic management strategies in acute care settings.

Although frailty has been linked to increased infection-related mortality in older ED patients [20,21]; its specific relationship with antibiotic-resistant organisms and treatment patterns remains poorly characterized. Traditional risk factors for antibiotic resistance, such as recent hospitalization, prior antibiotic use, and nursing home residence [22,23], may overlap with frailty; however, whether frailty confers an independent risk remains unclear. Clarifying this relationship is crucial for emergency department physicians because routine frailty assessment can inform empirical antibiotic selection, guide broad-spectrum coverage decisions, and support targeted antimicrobial stewardship interventions.

This study investigated the association between frailty and antibiotic-resistant bacterial infections in older adults presenting to the ED with fever. Frailty was assessed using the Clinical Frailty Scale (CFS), a well-validated nine-point tool ranging from very fit (CFS 1) to terminally ill (CFS 9) that enables rapid and reliable frailty assessment in emergency settings [24,25]. The specific aims of this study were to: (1) determine whether frailty severity, as measured by the CFS, is associated with increased rates of antibiotic-resistant isolates; (2) examine the influence of frailty on antibiotic treatment patterns, including duration of therapy and frequency of antibiotic modification; and (3) assess whether frailty independently predicts adverse clinical outcomes, including hospital admission, intensive care unit admission, and in-hospital mortality. We hypothesized that frail patients would demonstrate higher rates of antibiotic-resistant infections, require more complex antibiotic management, and experience worse clinical outcomes than non-frail patients.

## 2. Results

### 2.1. Study Cohort

**Figure 1:**
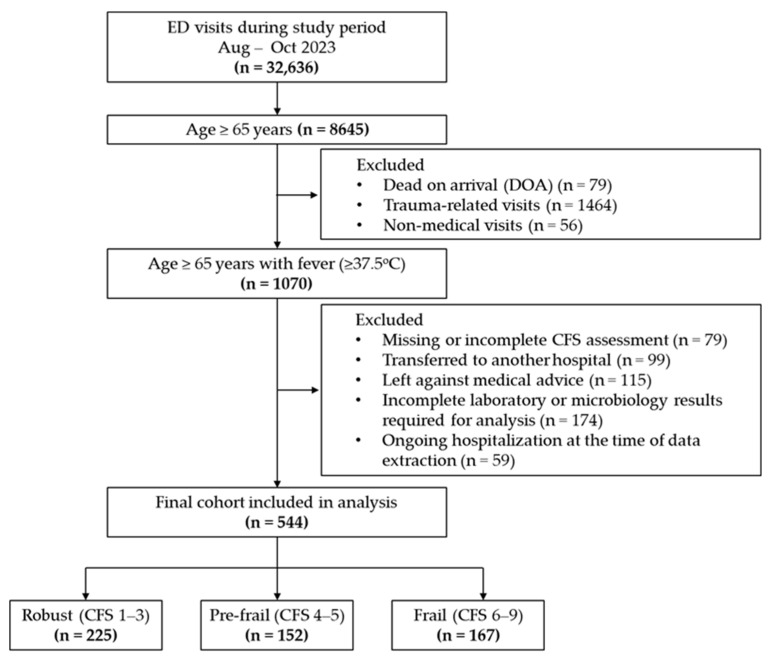
Study flow diagram of patient selection and frailty group classification.

A total of 544 older adults (≥65 years) presenting to the ED with fever were included in the analysis, as shown in Figure 1. Based on the CFS, patients were categorized as robust (*n* = 225, 41.3%), pre-frail (*n* = 152, 28.0%), or frail (*n* = 167, 30.7%). ED-initiated microbiological cultures (predominantly blood, urine, and sputum) were obtained for 329 patients (60.5%), of whom 180 (54.7%) had at least one positive culture result. Among treated patients with evaluable initial regimens, ceftriaxone remained the most common empirical antibiotic across frailty groups, whereas broader-spectrum agents such as piperacillin–tazobactam and meropenem were used more frequently in frail patients.

### 2.2. Baseline Characteristics by Frailty Group

Baseline characteristics, prior healthcare exposure, and clinical outcomes stratified by frailty group are summarized in Table 1. Demographic characteristics and vital signs varied significantly across frailty strata. Mean age increased progressively from the robust to the frail group (75.0 ± 6.9, 78.7 ± 7.1, and 80.4 ± 7.6 years, respectively; *p* < 0.001), whereas sex distribution remained comparable (male: 45.3%, 41.4%, and 41.3%; *p* = 0.656). Triage acuity, summarized as high acuity (KTAS levels 1–3), was similar across frailty groups (robust 89.3%, pre-frail 91.4%, frail 92.8%; *p* = 0.479).

Physiological parameters reflected increasing clinical severity with advancing frailty. Heart rate increased across frailty strata (95.4 ± 18.6, 98.3 ± 20.3, and 103.2 ± 21.7 beats/min; *p* < 0.001), while both systolic and diastolic blood pressure declined (SBP: 137.3 ± 27.2, 136.5 ± 28.9, and 126.2 ± 28.1 mmHg; *p* < 0.001; DBP: 74.8 ± 16.1, 73.0 ± 16.2, and 69.5 ± 17.3 mmHg; *p* = 0.008). Respiratory rate was higher in the frail group (20.2 ± 2.0, 20.3 ± 2.3, and 21.3 ± 3.4 breaths/min; *p* < 0.001), whereas body temperature remained similar across groups (38.2 ± 0.7, 38.4 ± 0.8, and 38.3 ± 0.7 °C; *p* = 0.314).

Laboratory findings demonstrated progressive inflammatory burden and nutritional deterioration with increasing frailty. WBC counts increased across strata (median [IQR]: 7.5 [5.5–10.2], 8.1 [6.4–13.1], and 8.4 [6.0–13.0] × 10^3^/μL; *p* < 0.001), while hemoglobin levels declined (12.7 [11.3–13.6], 12.1 [10.9–13.4], and 11.4 [10.0–13.0] g/dL; *p* < 0.001). C-reactive protein (CRP) levels increased progressively (1.9 [0.3–6.4], 2.9 [1.0–9.9], and 3.6 [1.0–13.5] mg/dL; *p* < 0.001), accompanied by declining serum albumin levels (3.9 [3.5–4.2], 3.7 [3.3–4.1], and 3.3 [3.0–3.7] g/dL; *p* < 0.001). Weighted comorbidity scores did not differ significantly across frailty groups (0.0 [0.0–1.0], 0.0 [0.0–1.0], and 1.0 [0.0–1.0]; *p* = 0.368).

Healthcare utilization patterns revealed a greater disease burden among frail patients. Prior hospitalization within 90 days increased across frailty strata (8.0%, 17.1%, and 20.4%, respectively; *p* = 0.001), as did prior systemic antibiotic use during the same period (8.0%, 15.1%, and 19.2%, respectively; *p* = 0.004). ED length of stay increased progressively (193.0 [142.0–269.0], 225.0 [155.5–298.5], and 275.0 [192.5–364.5] minutes; *p* < 0.001), paralleled by longer hospital length of stay (4.0 [0.0–11.0], 6.0 [0.0–11.0], and 9.0 [3.0–15.0] days; *p* < 0.001).

### 2.3. Microbiological Findings and Antibiotic-Related Metrics

The intensity and yield of microbiological testing increased with frailty severity (Table 2). The proportion of patients undergoing culture in the ED increased progressively across frailty strata (53.8%, 57.9%, and 71.9%; *p* = 0.001). Baseline characteristics and outcomes of culture-tested versus non-culture-tested patients are summarized in Appendix A (Table A1). To assess potential verification bias due to differential culture acquisition, we conducted sensitivity analyses including inverse probability weighting (IPW) based on the propensity of ED culture testing and a conservative assumption treating non-cultured patients as non-resistant; results were consistent with the primary analysis (Appendix A, Table A2). Among culture-tested patients, positivity rates increased similarly (46.3%, 53.4%, and 64.2%, respectively; *p* = 0.020). Bacteremia rates were comparable across groups (24.8%, 18.2%, and 24.2%, respectively; *p* = 0.480). In contrast, polymicrobial infections were significantly more frequent among frail patients (8.3%, 5.7%, and 20.0%, respectively; *p* = 0.002). Contamination rates showed no significant variation (19.8%, 17.0%, and 20.0%; *p* = 0.842).

**Figure 2:**
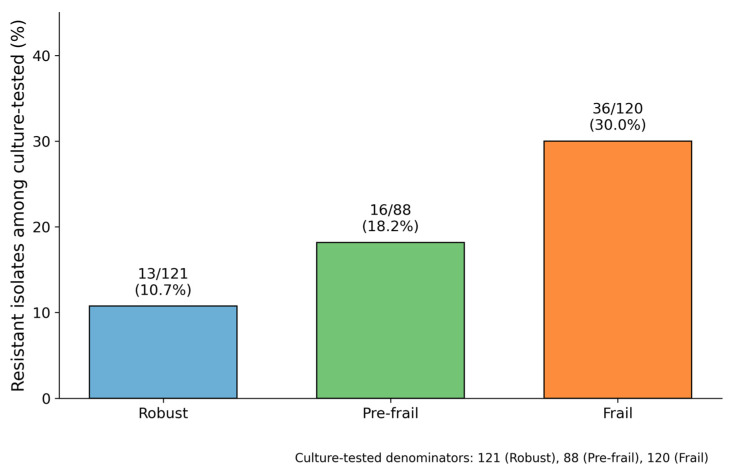
Proportion of antibiotic-resistant isolates among culture-tested patients by frailty group. Culture-tested patients were defined as those with any ED-initiated culture (robust, *n* = 121; pre-frail, *n* = 88; frail, *n* = 120). Values above bars indicate the number of resistant isolates per number of culture-tested patients (percentage).

Resistant isolates demonstrated a clear gradient across frailty categories and were detected in 10.7%, 18.2%, and 30.0% of culture-tested patients in the robust, pre-frail, and frail groups, respectively (*p* < 0.001) (Figure 2). Detailed resistant phenotype and organism distributions (including MRSA and Acinetobacter baumannii) are provided in Appendix A (Table A3 and Table A4). When stratified by pathogen category, the increasing trend with frailty was most evident for Enterobacterales, whereas resistant non-fermenters were infrequent and Gram-positive resistant isolates did not show a clear trend (Appendix A, Table A3).

Antibiotic management patterns reflected greater therapeutic complexity among frail patients (Table 2). The time from ED arrival to antibiotic administration did not differ significantly across frailty strata (median [IQR]: 180.0 [133.0–222.0], 166.2 [126.0–255.5], and 179.5 [139.2–267.8] minutes; *p* = 0.506). Frail patients experienced significantly higher rates of antibiotic regimen modification during hospitalization (68.1%) compared with robust (54.5%) and pre-frail (49.4%) patients (*p* = 0.020). Total antibiotic duration was also longer among frail patients (median, 11 [7,8,9,10,11,12,13,14,15,16,17,18] days) than among robust and pre-frail patients (both, 8 days; *p* = 0.005).

### 2.4. Multivariable Logistic Regression for Resistant Isolates

In multivariable analyses, frailty remained independently associated with detection of resistant isolates after sequential adjustment for potential confounders (Table 3). Among culture-tested patients (*n* = 329), frail patients demonstrated consistently higher odds of harboring resistant isolates compared with robust patients across all models: Model 1 (OR 3.10, 95% CI 1.38–6.99; *p* = 0.006), Model 2 (OR 3.25, 95% CI 1.43–7.38; *p* = 0.005), and Model 3 (OR 3.15, 95% CI 1.38–7.20; *p* = 0.006). In the fully adjusted model accounting for all relevant covariates (Model 4; *n* = 274), this association persisted with a substantial effect size (OR 2.84, 95% CI 1.15–7.04; *p* = 0.024).

### 2.5. Clinical Outcomes

Clinical outcomes progressively worsened with increasing frailty (Table 1). Hospital admission rates increased across frailty strata (54.2%, 63.8%, and 75.4%, respectively; *p* < 0.001), as did ICU admissions (17.8%, 27.6%, and 37.7%, respectively; *p* < 0.001). In-hospital mortality was significantly higher among frail patients than among robust patients (1.8%, 3.3%, and 7.2%, respectively; *p* = 0.021). In exploratory multivariable analyses using the same adjustment framework as Model 4, frailty was not independently associated with ICU admission or in-hospital mortality after adjustment (Appendix A, Table A5).

## 3. Discussion

This study demonstrated three critical findings regarding the relationship between frailty and antibiotic resistance in older adults presenting to the ED with fever. First, frailty was independently associated with antibiotic-resistant bacterial infections, with frail patients exhibiting nearly threefold higher odds of harboring resistant organisms than did robust patients, even after adjusting for traditional risk factors, including prior healthcare exposure and illness severity. Second, frailty was associated with increased therapeutic complexity, as manifested by higher rates of antibiotic regimen changes (68.1% vs. 54.5%, *p* = 0.020) and prolonged antibiotic courses (median 11 days vs. 8 days, *p* = 0.005). Third, frail patients experienced significantly worse clinical outcomes, including higher rates of hospital admission, ICU admission, and in-hospital mortality. Collectively, these findings suggest that routine frailty assessment in the ED may serve as a practical tool to identify older adults at high risk for resistant infections, thereby informing empirical antibiotic selection and guiding antimicrobial stewardship strategies in acute care settings.

The observed association between frailty and antibiotic-resistant infections likely reflects the convergence of biological vulnerability and exposure-related mechanisms. Frailty has been consistently linked to age-related immune dysregulation, including immunosenescence, which can blunt innate and adaptive antimicrobial responses and impair pathogen clearance [9,10,11,26]. In addition, frail older adults frequently exhibit inflammaging—a chronic, low-grade inflammatory state characterized by elevated circulating pro-inflammatory mediators such as IL-6, TNF-α, and CRP—which may further compromise effective immune surveillance and promote pathogen persistence [12,13]. Beyond host immunity, frailty is also associated with gut microbiome dysbiosis, characterized by reduced microbial diversity and shifts in community composition [14,15]. During physiological stress or following antimicrobial exposure, disruption of commensal microbial communities may weaken colonization resistance and facilitate the acquisition or overgrowth of antibiotic-resistant organisms [17,18,19]. Consistent with this framework, frail patients in our cohort demonstrated substantially higher rates of resistant isolates than did robust patients (30.0% vs. 10.7%), an association that persisted after adjustment for recent hospitalization and prior antibiotic use. This persistence suggests that frailty may confer biological susceptibility to resistant infections beyond healthcare exposure alone. Together, these findings extend prior ED-based studies linking frailty to adverse infection-related outcomes and reinforce the clinical value of incorporating frailty into risk stratification for older adults presenting with suspected infection [20,21,27,28].

Our findings have important implications for empirical antibiotic selection in older patients presenting to the ED with fever. The threefold increase in the odds of resistant infections among frail patients suggests that frailty assessment should be integrated into clinical decision-making algorithms for empirical antimicrobial therapy. Emergency physicians caring for older adults with frailty who present with fever should interpret frailty as a potential risk marker rather than a direct causal determinant of antibiotic resistance. In addition to biological vulnerability, frailty may also reflect cumulative healthcare exposure, prior colonization with resistant organisms, and functional dependency, all of which may contribute to the observed association. Therefore, frailty-informed decisions regarding empirical antibiotic coverage should be interpreted in the context of local antibiograms, infection source, and overall clinical presentation. This approach aligns with antimicrobial stewardship principles that emphasize timely, appropriate therapy while minimizing unnecessary broad-spectrum antibiotic use in lower-risk populations [29,30].

Accordingly, the antibiotic management patterns observed in this study warrant careful interpretation. Although the time to antibiotic initiation did not differ significantly across frailty groups (median, 179.5 min in frail vs. 180.0 min in robust patients; *p* = 0.506), frail patients experienced significantly higher rates of antibiotic regimen modification (68.1% vs. 54.5% in robust patients; *p* = 0.020) and longer total durations of antibiotic therapy (median, 11 days vs. 8 days in robust patients; *p* = 0.005). The comparable time to antibiotic administration across frailty groups likely reflects institutional protocols that prioritize early empirical therapy for suspected infection. However, frail older adults often present with atypical manifestations of infection and require more extensive diagnostic evaluation because of multimorbidity and diagnostic uncertainty [31,32]. In this context, the absence of delay in antibiotic initiation is reassuring; nevertheless, given that inadequate or delayed therapy for infections caused by antimicrobial-resistant organisms has been associated with adverse outcomes, these findings highlight an important opportunity for quality improvement [29]. The higher frequency of antibiotic regimen modification and prolonged treatment duration observed in frail patients likely reflects a combination of factors, including a greater burden of resistant organisms (30.0% in frail vs. 10.7% in robust patients; *p* < 0.001), the need for therapeutic escalation, slower clinical response due to impaired immune function, and an increased risk of complications requiring extended antimicrobial therapy. In addition, the high prevalence of polymicrobial infections among frail patients (20.0%), approximately 2.4-fold higher than that observed in robust patients (8.3%), further complicates therapy. Taken together, these findings underscore the need for close clinical monitoring and serial reassessment of antimicrobial therapy in frail patients, with early involvement of infectious disease specialists or antimicrobial stewardship teams when available.

Beyond resistant isolate detection, microbiological patterns differed across frailty strata. Polymicrobial infections were more than twofold higher in frail patients (20.0%) than in robust patients (8.3%; *p* = 0.002), whereas bacteremia (24.8%, 18.2%, and 24.2%; *p* = 0.480) and contaminant rates (19.8%, 17.0%, and 20.0%; *p* = 0.842) were similar across groups. The elevated polymicrobial burden likely reflects multiple contributors, including more recent healthcare exposures in frail patients, such as prior hospitalization (20.4%) and antibiotic use (19.2%) within 90 days, as well as frailty-associated alterations at the host–microbiome interface, including gut microbiome dysbiosis, which may facilitate colonization by diverse organisms [14,15,33]. The increased polymicrobial burden plausibly contributes to the therapeutic complexity observed in frail patients, including frequent antibiotic regimen changes and prolonged treatment duration. Notably, the similarity in bacteremia rates across frailty groups suggests that the frailty–resistance association is not solely driven by bloodstream infection severity but may reflect broader vulnerabilities related to age-related immune dysregulation [11,26], microbiome alterations, and cumulative healthcare exposures; comparable contaminant rates also indicate consistent culture collection quality across groups, supporting the internal validity of our microbiological comparisons.

The clinical outcomes observed in our study confirm and extend previous reports linking frailty to adverse outcomes in older ED patients with infections [27,28]. Frail patients experienced fourfold higher in-hospital mortality (7.2% vs. 1.8%), twice the ICU admission rate (37.7% vs. 17.8%), and significantly longer hospital stays than robust patients. These outcomes suggest that frailty represents a fundamental vulnerability that extends beyond antibiotic management alone, as adverse outcomes persist despite a comparable time to antibiotic initiation across groups and more intensive therapeutic efforts in frail patients, including higher rates of regimen changes and longer treatment duration. From a healthcare system perspective, the combination of higher ICU admission rates, longer hospital stays, and increased mortality in frail patients with fever highlights the substantial burden these patients place on acute care resources. The progressive increase in ED length of stay across frailty strata (193 min in robust vs. 275 min in frail patients) suggests that frail patients require more extensive ED-based evaluation and stabilization before disposition. Healthcare systems should consider specialized pathways or geriatric emergency medicine units to optimize care delivery for this high-risk population.

Our findings suggest that frailty assessment using the CFS provides clinically actionable information during ED evaluation of older adults with fever. The CFS offers distinct advantages for this purpose: unlike comprehensive geriatric assessment tools that require extensive time and specialized training, it can be rapidly administered by emergency personnel through brief structured interviews with patients or caregivers [25,34], making frailty screening scalable in busy ED environments. A clear dose–response relationship was observed, with resistance rates of 10.7%, 18.2%, and 30.0% across the robust, pre-frail, and frail categories, respectively, enabling meaningful risk stratification. For frail patients with fever, emergency physicians should prioritize microbiological culture collection given the high likelihood of resistant and polymicrobial infections; consider broader empirical coverage when clinical severity is warranted (accounting for MRSA, ESBL-producing organisms, and local resistance patterns); and develop protocols to reduce the time to antibiotic initiation while maintaining judicious stewardship through close monitoring and early de-escalation based on culture results. Integrating frailty assessment into ED workflows can also enhance risk communication and facilitate early specialty consultations when appropriate.

This study has several strengths, including its multicenter design across three university hospitals, granular clinical frailty categorization using a validated tool, and detailed capture of microbiological and antibiotic-related metrics. Our sequential multivariable modeling approach systematically evaluated the independence of the frailty–resistance association after controlling for established risk factors. However, this study had some limitations. First, the retrospective observational design limits causal inference; therefore, the observed association between frailty and antibiotic resistance should not be interpreted as causal. Second, detection of resistant isolates depends on culture testing, and culture acquisition may have been influenced by clinician judgment. Because cultures were not obtained universally, selection bias in culture-dependent outcomes cannot be fully excluded. Although we addressed this by restricting culture-dependent outcomes to patients who underwent culture testing (71.9% in frail vs. 53.8% in robust patients), differences between culture-tested and non-culture-tested patients may have introduced residual selection bias. Third, Model 4 included additional laboratory covariates and therefore had a smaller sample size for complete-case analysis (*n* = 274), which may have reduced the precision of effect estimates. Fourth, standardized severity-of-illness scores (e.g., SOFA or APACHE) could not be calculated because key components were not uniformly available in this retrospective multicenter ED dataset. We therefore adjusted for routinely available objective severity markers at presentation (including lactate) and provided additional context using hypotension and a pragmatic shock proxy across frailty strata (Appendix A, Table A6). In addition, because only a limited standardized set of comorbidity variables was harmonized across sites, we used a weighted comorbidity score tailored to conditions relevant to antimicrobial resistance risk, and a full Charlson Comorbidity Index could not be computed. Fifth, antibiotic-related metrics may reflect both disease complexity and variations in institutional practice that cannot be fully disentangled in an observational design. Finally, our three-month study period and single metropolitan South Korean setting may limit generalizability to other healthcare systems with different antibiotic prescribing patterns or population demographics.

## 4. Materials and Methods

### 4.1. Study Design and Setting

This retrospective multicenter cohort study was conducted at three university hospitals in a metropolitan area of South Korea between 1 August and 31 October 2023. The study was approved by the Institutional Review Boards of all participating institutions, and the requirement for informed consent was waived because of the retrospective nature of the study.

### 4.2. Study Population

We included patients aged ≥65 years who presented to the ED with fever, defined as a tympanic temperature ≥37.5 °C at triage. Patients were excluded if they were dead on arrival, had trauma-related or non-medical visits, were transferred to another hospital, left against medical advice, had missing or incomplete CFS assessment, had incomplete laboratory or microbiology results required for the prespecified analyses, or were still hospitalized at the time of data extraction (Figure 1).

### 4.3. Frailty Assessment

Frailty was assessed using the CFS, a validated 9-point scale ranging from 1 (very fit) to 9 (terminally ill). At the time of ED presentation, CFS scores were assigned by trained emergency department personnel across participating centers using a standardized approach based on structured patient/caregiver interviews and chart review of baseline function prior to the acute illness. The prospectively recorded CFS scores were retrospectively extracted from electronic medical records for analysis. Although formal inter-rater reliability testing across centers was not performed in this retrospective dataset, participating investigators periodically reviewed collected data and discussed representative cases to promote consistency in CFS scoring across sites. For the primary analysis, patients were categorized into three groups based on established CFS thresholds: robust (CFS 1–3), pre-frail (CFS 4–5), and frail (CFS 6–9). This three-tier classification has been widely used in geriatric emergency medicine research and allows the evaluation of dose–response relationships between frailty severity and clinical outcomes.

### 4.4. Primary Outcome

The primary outcome was the detection of antibiotic-resistant bacterial isolates in patients who underwent microbiological culture testing during ED visits. Antibiotic-resistant bacteria were defined according to standard clinical microbiology criteria and included methicillin-resistant *Staphylococcus aureus* (MRSA), vancomycin-resistant *Enterococci* (VRE), extended-spectrum beta-lactamase (ESBL)-producing *Enterobacteriaceae*, carbapenem-resistant *Enterobacteriaceae* (CRE), multidrug-resistant Pseudomonas aeruginosa, and Acinetobacter baumannii. Multidrug resistance was defined based on routine clinical microbiology reporting and antimicrobial susceptibility testing performed at each participating institution, and susceptibility interpretations were made according to the Clinical and Laboratory Standards Institute (CLSI, Wayne, PA, USA) performance standards [35]. Because this was a retrospective multicenter study, detailed information on specific culture media and platform-specific AST panels/drug lists was not uniformly retrievable across institutions. The denominator for the primary outcome analysis included only patients who had at least one culture performed (blood, urine, sputum, and other clinically indicated cultures) during their ED stay, because resistant organisms could not be detected in patients who did not undergo culture testing. Cultures obtained after hospital admission were not included in the outcome assessment.

### 4.5. Data Collection

Trained research personnel extracted data from electronic medical records. The demographic variables included age and sex. Vital signs at ED arrival (temperature, heart rate, blood pressure, respiratory rate, and oxygen saturation) and triage acuity (Korean Triage and Acuity Scale [KTAS] level) were recorded [36].

Laboratory values obtained upon ED arrival included white blood cell (WBC) count, hemoglobin, platelet count, C-reactive protein (CRP), serum creatinine, albumin, and lactate levels. CRP values were measured using different assays across hospitals: one hospital reported values in mg/L (reference range ≤ 5 mg/L), while two hospitals reported values in mg/dL (reference range ≤ 0.3 mg/dL). To ensure comparability, the CRP values reported in mg/L were converted to mg/dL by dividing by 10. Lactic acid measurements varied according to the hospital assay methodology; values were standardized to mmol/L using a conversion factor of 9.008 where necessary.

The comorbidity burden was quantified using a weighted comorbidity score based on four conditions with established associations with antimicrobial resistance and healthcare-associated infections: diabetes mellitus (1 point), chronic kidney disease (2 points), chronic obstructive pulmonary disease or lung cancer (1 point), and congestive heart failure or cardiovascular disease (1 point). The weighting scheme was adapted from the Charlson Comorbidity Index [37]. Prior healthcare exposure was defined as any hospitalization or systemic antibiotic use within 90 days before the index ED visit, consistent with the established definitions of healthcare-associated infection risk [38].

Microbiological data included whether cultures were performed, culture results (positive vs. negative), source of the index culture, specific isolated organisms, and antibiotic susceptibility patterns. ED culture acquisition was performed as part of routine clinical care based on suspected infection source and clinician judgment rather than a study-mandated standardized protocol. Quantitative colony counts (CFU/mL) were not consistently reported or retrievable across specimen types and institutions in this retrospective multicenter dataset and were therefore not analyzed. Antibiotic-related variables included (1) time from ED arrival to first antibiotic administration (ED-to-antibiotic time), calculated in minutes from documented ED arrival to the time of the first antibiotic dose; (2) antibiotic change, defined as any modification to the initial antibiotic regimen during the hospital stay, including escalation, de-escalation, switching, adding, or discontinuing antimicrobial agents; and (3) total duration of antibiotic therapy, recorded as the number of days from initiation to discontinuation of all antimicrobial agents during the index hospitalization. Microbiological outcomes and antibiotic-related metrics were summarized by frailty group. Clinical outcomes included hospital admission, ICU admission, in-hospital mortality, ED length of stay, and length of hospital stay among admitted patients.

### 4.6. Statistical Analysis

Continuous variables were presented as mean ± standard deviation for normally distributed data or median (interquartile range) for skewed distributions. Categorical variables were presented as frequencies and percentages. Baseline characteristics were compared across the three frailty groups (robust, pre-frail, and frail) using one-way analysis of variance (ANOVA) or the Kruskal–Wallis test for continuous variables and the chi-squared test or Fisher’s exact test for categorical variables, as appropriate.

The proportion of patients undergoing culture testing in the ED was calculated using the full cohort denominator within each frailty group, and culture-dependent outcomes were summarized among the culture-tested patients. For primary outcome analysis, we calculated the proportion of patients with antibiotic-resistant isolates among those who underwent culture testing, stratified by frailty. The Cochran–Armitage trend test was used to assess the dose–response relationship across frailty categories [39,40].

Multivariable logistic regression models were constructed to examine the independent association between frailty and antibiotic-resistant infections in culture-tested patients. We used a sequential modeling approach to evaluate the robustness of the frailty effect after progressive adjustment for potential confounders.

- Model 1 adjusted for demographic factors (age and sex) and culture positivity.
- Model 2 additionally adjusted for comorbidity burden (weighted comorbidity score).
- Model 3 was further adjusted for prior healthcare exposure (hospitalization and antibiotic use within 90 days).
- Model 4 additionally adjusted for illness severity markers at presentation (WBC, CRP, serum creatinine, albumin, and lactate levels) and was conducted as a complete-case analysis among culture-tested patients with available laboratory data (*n* = 274). Baseline characteristics of included versus excluded patients are provided in Appendix A (Table A7).

Multicollinearity was assessed using variance inflation factors (VIFs) for predictors included in Model 4; VIF values indicated no concerning multicollinearity (maximum VIF = 3.39) (Appendix A, Table A8).

The results are presented as adjusted odds ratios (ORs) with 95% confidence intervals (CIs). Statistical significance was defined as a two-tailed *p*-value < 0.05. All analyses were performed using SPSS (version 27.0; IBM Corp., Armonk, NY, USA) and R version 4.3.0 (R Foundation for Statistical Computing, Vienna, Austria).

## 5. Conclusions

Frailty was independently associated with the detection of antibiotic-resistant isolates among culture-tested older adults presenting to the ED with fever, even after accounting for traditional risk factors. Frail patients also experienced greater therapeutic complexity and worse clinical outcomes. These findings suggest that frailty assessment may serve as a useful clinical risk marker to support risk stratification and help inform empirical antibiotic decision-making and antimicrobial stewardship, although the observed association may also reflect cumulative healthcare exposure, prior colonization, and functional dependency. The CFS is a practical tool for rapid frailty screening in busy ED settings; however, given the retrospective design and that cultures were obtained in a subset of patients, prospective studies with standardized microbiology reporting are warranted to confirm these findings.

## Figures and Tables

**Table 1:** Baseline characteristics and clinical outcomes by frailty group.

Characteristic		Robust	Pre-Frail	Frail	*p*-Value
		(*n* = 225)	(*n* = 152)	(*n* = 167)	
Age, years ^a^		75.0 ± 6.9	78.7 ± 7.1	80.4 ± 7.6	**<0.001**
Sex; Male ^c^		102 (45.3)	63 (41.4)	69 (41.3)	0.656
High acuity (KTAS 1–3) ^c^		201 (89.3)	139 (91.4)	155 (92.8)	0.479
Vital Signs ^a^					
	SBP, mmHg	137.3 ± 27.2	136.5 ± 28.9	126.2 ± 28.1	**<0.001**
	DBP, mmHg	74.8 ± 16.1	73.0 ± 16.2	69.5 ± 17.3	**0.008**
	Heart rate, beats/min	95.4 ± 18.6	98.3 ± 20.3	103.2 ± 21.7	**<0.001**
	Respiratory rate, /min	20.2 ± 2.0	20.3 ± 2.3	21.3 ± 3.4	**<0.001**
	Body temperature, °C	38.2 ± 0.7	38.4 ± 0.8	38.3 ± 0.7	0.314
Laboratory Findings ^b^					
	WBC, ×10^3^/µL	7.5 [5.5–10.2]	8.1 [6.4–13.1]	8.4 [6.0–13.0]	**<0.001**
	Hemoglobin, g/dL	12.7 [11.3–13.6]	12.1 [10.9–13.4]	11.4 [10.0–13.0]	**<0.001**
	Platelet count, ×10^3^/µL	189.0 [154.8–245.0]	188.0 [149.0–237.0]	194.0 [143.0–264.8]	0.575
	CRP, mg/dL	1.9 [0.3–6.4]	2.9 [1.0–9.9]	3.6 [1.0–13.5]	**<0.001**
	Creatinine, mg/dL	0.9 [0.7–1.1]	0.9 [0.7–1.2]	1.0 [0.7–1.5]	0.298
	Albumin, g/dL	3.9 [3.5–4.2]	3.7 [3.3–4.1]	3.3 [3.0–3.7]	**<0.001**
	Lactate, mmol/L	1.7 [1.3–2.4]	1.8 [1.2–2.3]	2.0 [1.3–2.8]	0.055
Weighted comorbidity score (Charlson-like) ^b^		0.0 [0.0–1.0]	0.0 [0.0–1.0]	1.0 [0.0–1.0]	0.368
Prior hospitalization within 90 days ^c^		18 (8.0)	26 (17.1)	34 (20.4)	**0.001**
Prior systemic antibiotic use within 90 days, n (%)		18 (8.0)	23 (15.1)	32 (19.2)	**0.004**
ED length of stay, min ^b^		193.0 [142.0–269.0]	225.0 [155.5–298.5]	275.0 [192.5–364.5]	**<0.001**
Hospital length of stay, days ^b^		4.0 [0.0–11.0]	6.0 [0.0–11.0]	9.0 [3.0–15.0]	**<0.001**
Clinical outcomes ^c^					
	Hospital admission	122 (54.2)	97 (63.8)	126 (75.4)	**<0.001**
	ICU admission	40 (17.8)	42 (27.6)	63 (37.7)	**<0.001**
	In-hospital mortality	4 (1.8)	5 (3.3)	12 (7.2)	**0.021**

^a^ mean ± SD; ^b^ median [IQR]; ^c^ *n* (%). Bold font indicates statistical significance (*p* < 0.05). Abbreviations: KTAS, Korean Triage and Acuity Scale; SBP, systolic blood pressure; DBP, diastolic blood pressure; WBC, white blood cell count; CRP, C-reactive protein; ED, emergency department; ICU, intensive care unit.

**Table 2:** Microbiological findings and antibiotic-related metrics by frailty group.

Variable		Robust	Pre-Frail	Frail	*p*-Value
		(*n* = 225)	(*n* = 152)	(*n* = 167)	
Microbiology (ED-initiated cultures)					
Any culture performed in ED ^a^		121 (53.8)	88 (57.9)	120 (71.9)	**0.001**
	Any culture positive among culture-tested ^a^	56/121 (46.3)	47/88 (53.4)	77/120 (64.2)	**0.020**
	Bacteremia among culture-tested ^a^	30/121 (24.8)	16/88 (18.2)	29/120 (24.2)	0.480
	Polymicrobial infection among culture-tested ^a^	10/121 (8.3)	5/88 (5.7)	24/120 (20.0)	**0.002**
	Contaminant flag among culture-tested ^a^	24/121 (19.8)	15/88 (17.0)	24/120 (20.0)	0.842
	Any resistant isolate among culture-tested ^a^	13/121 (10.7)	16/88 (18.2)	36/120 (30.0)	**<0.001**
Antibiotic-related metrics (treated subset)					
ED to antibiotic time, min ^b^		180.0 [133.0–222.0]	166.2 [126.0–255.5]	179.5 [139.2–267.8]	0.506
Antibiotic change during hospitalization ^a,^*		54/99 (54.5)	41/83 (49.4)	79/116 (68.1)	**0.020**
Antibiotic duration, days ^b^		8 [5,6,7,8,9,10,11,12,13,14]	8 [5,6,7,8,9,10,11]	11 [7,8,9,10,11,12,13,14,15,16,17,18]	**0.005**

^a^ n (%); ^b^ median [IQR]. Culture-related outcomes (any positive culture, bacteremia, polymicrobial infection, contaminant flag, and resistant isolate) were calculated among culture-tested patients (i.e., those with any ED-initiated culture). Antibiotic-related metrics were assessed in the treated subset with available data; denominators may vary by variable. * Antibiotic change during hospitalization was assessed among patients who received antibiotics and were hospitalized, restricted to those with retrievable inpatient antibiotic administration records (evaluable denominator). *p*-values represent between-group comparisons across frailty groups. Trends across ordered frailty categories were additionally assessed using the Cochran–Armitage trend test (*p* for trend: culture performed < 0.001; culture positive 0.005; bacteremia 0.906; polymicrobial infection 0.005; contaminant 0.975; resistant isolate < 0.001). Bold font indicates statistical significance (*p* < 0.05). Abbreviations: ED, emergency department; IQR, interquartile range.

**Table 3:** Logistic regression models for antibiotic-resistant isolates among culture-tested patients by frailty group.

Variable (vs. Robust)	OR (95% CI)	*p*-Value
Model 1 (adjusted for age, sex, and culture positivity)		
Pre-frail	2.09 (0.89–4.90)	0.088
Frail	3.10 (1.38–6.99)	**0.006**
Model 2 (Model 1 + comorbidity burden)		
Pre-frail	2.25 (0.95–5.31)	0.066
Frail	3.25 (1.43–7.38)	**0.005**
Model 3 (Model 2 + prior healthcare exposure)		
Pre-frail	2.18 (0.92–5.18)	0.078
Frail	3.15 (1.38–7.20)	**0.006**
Model 4 (Model 3 + illness severity markers at presentation) *		
Pre-frail	2.03 (0.78–5.24)	0.145
Frail	2.84 (1.15–7.04)	**0.024**

Odds ratios (ORs) with 95% confidence intervals (CIs) from logistic regression are shown; robust was the reference group. Analyses were restricted to culture-tested patients (*n* = 329). * Model 4 was based on complete cases (*n* = 274). Bold font indicates statistical significance (*p* < 0.05). Abbreviations: OR, odds ratio; CI, confidence interval.

## Data Availability

The data presented in this study are available on request from the corresponding author due to privacy and ethical restrictions.

## Abbreviations

The following abbreviations are used in this manuscript:

CFS	Clinical Frailty Scale
ED	Emergency Department
ICU	Intensive Care Unit
KTAS	Korean Triage and Acuity Scale
WBC	White Blood Cell count
CRP	C-Reactive Protein
SBP	Systolic Blood Pressure
DBP	Diastolic Blood Pressure
MRSA	Methicillin-Resistant *Staphylococcus aureus*
VRE	Vancomycin-Resistant *Enterococci*
ESBL	Extended-Spectrum Beta-Lactamase
CRE	Carbapenem-Resistant *Enterobacteriaceae*

## Appendix A

**Table A1:** Comparison of culture-tested vs. non-culture-tested patients.

Variable	Culture-Tested	Non-Culture-Tested	*p*-Value
	(*n* = 329)	(*n* = 215)	
Age, years	78.2 ± 7.5	76.9 ± 7.5	**0.041**
Male; sex	150 (45.6)	84 (39.1)	0.157
CFS group (robust/pre-frail/frail)	121 (36.8)/88 (26.7)/120 (36.5)	104 (48.4)/64 (29.8)/47 (21.9)	**0.001**
Temperature, °C	38.4 ± 0.7	38.1 ± 0.7	**<0.001**
Systolic BP, mmHg	130.2 ± 27.2	138.9 ± 29.4	**<0.001**
Prior hospitalization within 90 days	69 (21.0)	9 (4.2)	**<0.001**
Prior systemic antibiotic use within 90 days	65 (19.8)	8 (3.7)	**<0.001**
Hospital admission	267 (81.2)	78 (36.3)	**<0.001**
ICU admission	105 (31.9)	40 (18.6)	**<0.001**
In-hospital mortality	15 (4.6)	6 (2.8)	0.413

Values are mean ± SD or n (%). *p*-values reflect between-group comparisons. Bold font indicates statistical significance (*p* < 0.05).

**Table A2:** Sensitivity analyses addressing selection in culture testing.

Analysis	Pre-Frail vs. Robust OR (95% CI)	*p*-Value	Frail vs. Robust OR (95% CI)	*p*-Value
Primary analysis (culture-tested subset)	1.52 (0.62–3.77)	0.362	2.78 (1.19–6.45)	**0.018**
IPW sensitivity (weighted by propensity of ED culture testing)	1.69 (0.66–4.34)	0.272	3.14 (1.26–7.83)	**0.014**
Conservative sensitivity (non-cultured assumed non-resistant)	1.53 (0.69–3.37)	0.295	3.43 (1.68–6.98)	**<0.001**

IPW was based on a propensity model for ED culture testing using baseline covariates (age, sex, frailty group, triage acuity, vital signs, arrival route, comorbidity score, and prior healthcare exposure). Bold font indicates statistical significance (*p* < 0.05).

**Table A3:** Resistant phenotypes and key organisms among culture-tested patients, by frailty group.

Category	Overall	Robust	Pre-Frail	Frail
	(*n* = 329)	(*n* = 121)	(*n* = 88)	(*n* = 120)
MRSA	0/329 (0.0)	0/121 (0.0)	0/88 (0.0)	0/120 (0.0)
VRE	0/329 (0.0)	0/121 (0.0)	0/88 (0.0)	0/120 (0.0)
ESBL-producing Enterobacterales	10/329 (3.0)	4/121 (3.3)	1/88 (1.1)	5/120 (4.2)
CRE/carbapenemase-positive Enterobacterales	1/329 (0.3)	0/121 (0.0)	0/88 (0.0)	1/120 (0.8)
Resistant *Pseudomonas aeruginosa*	6/329 (1.8)	0/121 (0.0)	2/88 (2.3)	4/120 (3.3)
Resistant *Acinetobacter baumannii*	1/329 (0.3)	1/121 (0.8)	0/88 (0.0)	0/120 (0.0)
MR-CoNS (labeled)	1/329 (0.3)	0/121 (0.0)	0/88 (0.0)	1/120 (0.8)
Pathogen category (culture-tested denominator)				
Resistant Enterobacterales (category)	40/329 (12.2)	6/121 (5.0)	10/88 (11.4)	24/120 (20.0)
Resistant non-fermenters (category)	9/329 (2.7)	1/121 (0.8)	3/88 (3.4)	5/120 (4.2)
Resistant Gram-positive organisms (category)	9/329 (2.7)	4/121 (3.3)	3/88 (3.4)	2/120 (1.7)
Fungal (*Candida* spp.)	1/329 (0.3)	1/121 (0.8)	0/88 (0.0)	0/120 (0.0)
Other organisms (not categorized)	7/329 (2.1)	1/121 (0.8)	1/88 (1.1)	5/120 (4.2)

n/N (%). N indicates the number of culture-tested patients in each frailty group (overall 329; robust 121; pre-frail 88; frail 120). Resistant phenotypes/organisms were abstracted from routine clinical microbiology report labels and antimicrobial susceptibility interpretations. “CRE/carbapenemase-positive” reflects a report-level label captured in the extracted microbiology fields. Pathogen categories are not mutually exclusive; polymicrobial entries may contribute to more than one category. Abbreviations: CRE, carbapenem-resistant Enterobacterales; ESBL, extended-spectrum beta-lactamase; MR-CoNS, methicillin-resistant coagulase-negative *Staphylococci*; MRSA, methicillin-resistant *Staphylococcus aureus*; VRE, Vancomycin-resistant *Enterococci*.

**Table A4:** Distribution of organisms among patients with resistant isolates, by frailty group.

Organism	Overall	Robust	Pre-Frail	Frail
	(*n* = 65)	(*n* = 13)	(*n* = 16)	(*n* = 36)
*Escherichia coli*	35 (53.8)	6 (46.2)	9 (56.2)	20 (55.6)
*Pseudomonas aeruginosa*	6 (9.2)	0 (0.0)	2 (12.5)	4 (11.1)
*Enterococcus faecalis*	4 (6.2)	1 (7.7)	1 (6.2)	2 (5.6)
*Enterococcus faecium*	4 (6.2)	0 (0.0)	1 (6.2)	3 (8.3)
Coagulase-negative *Staphylococcus* spp.	4 (6.2)	2 (15.4)	2 (12.5)	0 (0.0)
*Staphylococcus aureus*	3 (4.6)	2 (15.4)	0 (0.0)	1 (2.8)
*Klebsiella pneumoniae*	3 (4.6)	0 (0.0)	0 (0.0)	3 (8.3)
*Enterobacter* spp.	2 (3.1)	0 (0.0)	1 (6.2)	1 (2.8)
*Proteus mirabilis*	2 (3.1)	0 (0.0)	0 (0.0)	2 (5.6)
*Stenotrophomonas maltophilia*	2 (3.1)	0 (0.0)	1 (6.2)	1 (2.8)
*Acinetobacter baumannii*	1 (1.5)	1 (7.7)	0 (0.0)	0 (0.0)
*Candida* spp.	1 (1.5)	1 (7.7)	0 (0.0)	0 (0.0)
Other organisms	1 (1.5)	1 (7.7)	0 (0.0)	0 (0.0)

n (%). Percentages were calculated using the number of patients with resistant isolates as the denominator in each frailty group (overall 65; robust 13; pre-frail 16; frail 36). Polymicrobial entries may contribute to more than one organism category.

**Table A5:** Exploratory multivariable analyses for ICU admission and in-hospital mortality.

Outcome (Comparison vs. Robust)		Adjusted OR (95% CI)	*p*-Value
ICU admission			
	Pre-frail	1.27 (0.68–2.37)	0.455
	Frail	1.26 (0.68–2.34)	0.466
In-hospital mortality			
	Pre-frail	1.09 (0.20–5.85)	0.919
	Frail	0.72 (0.14–3.73)	0.699

Exploratory models used the same covariate framework as Model 4 (age, sex, weighted comorbidity score, prior hospitalization within 90 days, prior systemic antibiotic use within 90 days, WBC, CRP, creatinine, albumin, and lactate) and were conducted as complete-case analyses (*n* = 373).

**Table A6:** Severity context across frailty strata in culture-tested patients.

Marker	Robust	Pre-Frail	Frail
Hypotension at triage (SBP < 90 mmHg)	4/121 (3.3)	4/88 (4.5)	11/120 (9.2)
Shock proxy ^†^ (SBP < 90 or MAP < 65 and lactate ≥ 2 mmol/L) ^‡^	3/99 (3.0)	3/71 (4.2)	9/106 (8.5)

^†^ Defined using available vital signs and lactate. ^‡^ Calculated among patients with lactate measured (denominators shown).

**Table A7:** Comparison of culture-tested patients included vs. excluded from Model 4 due to missing laboratory data.

Variable	Included in Model 4	Excluded	*p*-Value
	(*n* = 274)	(*n* = 55)	
Age, years	78.5 ± 7.5	76.7 ± 7.6	0.098
Male; sex	129 (47.1)	21 (38.2)	0.289
CFS group (robust/pre-frail/frail)	99/70/105	22/18/15	0.272
Weighted comorbidity score	0.94 ± 1.05	0.85 ± 1.08	0.593
Prior hospitalization within 90 days	60 (21.9)	9 (16.4)	0.460
Prior systemic antibiotic use within 90 days	58 (21.2)	7 (12.7)	0.212
Any culture positive	158 (57.7)	22 (40.0)	**0.024**
Any resistant isolate (primary outcome)	57 (20.8)	8 (14.5)	0.380
ICU admission	101 (36.9)	4 (7.3)	**<0.001**
In-hospital mortality	13 (4.7)	2 (3.6)	0.996

Values are mean ± SD or *n* (%). *p*-values reflect between-group comparisons. Bold font indicates statistical significance (*p* < 0.05).

**Table A8:** Variance inflation factors (VIFs) for predictors in Model 4.

Predictor	VIF
Age	1.18
Male; Sex	1.09
Culture positivity	1.17
Weighted comorbidity score	1.16
Prior hospitalization within 90 days	3.34
Prior systemic antibiotic use within 90 days	3.39
White blood cell count	1.25
C-reactive protein	1.45
Serum creatinine	1.18
Serum albumin	1.54
Serum lactate	1.11
Frailty (pre-frail vs. robust)	1.40
Frailty (frail vs. robust)	1.69

VIFs were calculated for all predictors included in Model 4. No VIF exceeded 5 (maximum 3.39), indicating no concerning multicollinearity.
